# Effect of lisdexamfetamine dimesylate on sleep in adults with attention-deficit/hyperactivity disorder

**DOI:** 10.1186/1744-9081-5-34

**Published:** 2009-08-03

**Authors:** Lenard A Adler, David Goodman, Richard Weisler, Mohamed Hamdani, Thomas Roth

**Affiliations:** 1 Department of Psychiatry and Child and Adolescent Psychiatry, New York University School of Medicine, NY, USA; 2 Department of Psychiatry and Behavioral Sciences, Johns Hopkins University School of Medicine, Baltimore, MD, USA; 3 Department of Psychiatry, Duke University Medical Center, Durham, NC, USA; 4 University of North Carolina at Chapel Hill, Chapel Hill, NC, USA; 5 Biostatistics, Shire Pharmaceuticals Inc., Wayne, PA, USA; 6 Sleep Disorders and Research Center, Henry Ford Health System, Detroit, MI, USA

## Abstract

**Background:**

Sleep problems are common in adults with attention-deficit/hyperactivity disorder (ADHD). This analysis aimed to evaluate the impact of lisdexamfetamine dimesylate (LDX) on sleep quality in adults with ADHD.

**Methods:**

This 4-week, phase 3, double-blind, forced-dose escalation study of adults aged 18 to 55 years with ADHD randomized participants to receive placebo (n = 62), or 30 (n = 119), 50 (n = 117), or 70 (n = 122) mg/d LDX, taken once a day in the morning. The self-rated Pittsburgh Sleep Quality Index (PSQI) was administered at baseline and at week 4 to assess sleep quality. The PSQI global score assesses 7 sleep components (subjective sleep quality, sleep latency, sleep duration, habitual sleep efficiency, sleep disturbances, use of sleeping medications, and daytime dysfunction) each scored from 0 (no difficulty) to 3 (severe difficulty).

**Results:**

The mean baseline PSQI global score was 5.8 for LDX and 6.3 for placebo (*P *= .19) indicating poor overall sleep quality. At endpoint, least squares (LS) mean change from baseline was -0.8 for LDX vs -0.5 for placebo (*P *= .33). The daytime functioning component showed significant improvement in LS mean change at endpoint for LDX compared with placebo (LDX -0.4 vs placebo 0.0, *P *= .0001). LS mean changes for the other 6 PSQI components did not significantly differ from placebo. Sleep-related treatment-emergent adverse events with an incidence ≥2% in the active treatment and placebo groups, respectively, were insomnia (19.3% and 4.8%), initial insomnia (5.0% and 3.2%), middle insomnia (3.6% and 0%), sleep disorder (0.6% and 3.2%), somnolence (0.3% and 3.2%), and fatigue (4.7% and 4.8%), and were generally mild or moderate in severity.

**Conclusion:**

For most subjects, LDX was not associated with an overall worsening of sleep quality and significantly improved daytime functioning in adults with ADHD.

**Trial Registration:**

clinicaltrials.gov Identifier: NCT00334880

## Background

Attention deficit/hyperactivity disorder (ADHD), characterized by a pattern of inattention and/or hyperactivity, is one of the most prevalent psychiatric disorders worldwide [1]. Often diagnosed in childhood, ADHD is thought to affect as many as 8% of children globally [2-4]. Symptoms of ADHD persist into adolescence and adulthood for approximately two-thirds of childhood cases [5,6]. The prevalence of ADHD in adults in the United States is estimated to be 4.4% [7].

Subjective reports of sleep problems are frequent among both adult and pediatric patients with ADHD [8,9]. The relationship between sleep and ADHD is complex and appears to be bidirectional in that ADHD affects sleep patterns while sleep patterns can impact ADHD symptoms [10]. The mechanisms by which ADHD can disturb sleep have not been fully elucidated but may involve changes in noradrenergic and/or dopaminergic systems. Furthermore, anatomical and functional regions of the central nervous system responsible for ADHD symptoms and those involved in sleep regulation seemingly overlap [10]. Patient reports of disturbed sleep are also significantly associated with their current ADHD symptom severity. A study by Kass et al demonstrated that daytime sleepiness and insomnia were predictors of increased ADHD symptomatology as assessed by the Adult Behavior Checklist in young adults [11].

Sleep disturbances in ADHD may be a manifestation of ADHD, itself, but may also result from medications used to treat ADHD [10,12]. Sleep disturbances may also be secondary to comorbid conditions [10,12]. It is possible that sleep disturbances secondary to ADHD and/or pharmacotherapy may also be contributing factors in driving-related problems known to be associated with ADHD [13].

The increased prevalence of sleep disturbances in adults with ADHD may also be secondary to other behaviors or conditions associated with ADHD. For instance, cigarette use, which is significantly more common in adolescents and adults with ADHD than in healthy controls, has been shown to independently result in sleep disturbances, including, but not limited to, less total sleep time, extended sleep onset latency, and dissatisfaction with sleep quality [5,14,15]. Caffeine intake is well known to adversely affect sleep [16], but at the same time, may be used to some extent for self-medication in adults with ADHD [17]. There also appears to be an association between obesity and ADHD [18]. Obesity is an important risk factor for obstructive sleep apnea-hypopnea syndrome, a disorder associated with sleep fragmentation and frequent nocturnal arousals leading to daytime sleepiness [19].

Although subjective reports of sleep disturbances in adults with ADHD are frequent in the clinical literature, to date, there is scant direct evidence of the occurrence of specific sleep disorders in adults with ADHD [20]. Wagner et al noted that ADHD symptoms were more common (26%) in a cohort of subjects with restless leg syndrome (RLS) than those with primary insomnia (6%) or normal controls (5%) suggesting a link between ADHD and RLS in adults [21]. A recent small (N = 6) pilot study found polysomnographic evidence of sleep-disordered breathing to support subjective reports of sleep disturbances in adult subjects with carefully diagnosed ADHD [22]. Another case study further supports the notion that ADHD and sleep-disordered breathing in the form of obstructive sleep apnea may be associated [23]. A number of studies have investigated the sleep patterns of subjects with ADHD compared with healthy adults. Schredl et al demonstrated that adults with ADHD rated their sleep quality and feeling of being refreshed in the morning much lower than healthy controls; sleep latency and nocturnal awakenings were marginally significantly different from controls [24]. The cross-sectional study did not demonstrate any effect of medication on sleep parameters as assessed by patient-completed questionnaires. Using a combination of sleep log and actigraph results, Kooij and colleagues reported that adults with ADHD demonstrated significantly poorer sleep quality and higher nocturnal motor activity than their matched controls but did not differ in reported total time in bed, sleep latency, or number of awakenings [25].

Along with Surman et al above [22], other studies have provided further objective (eg, polysomnography or sleep actigraphy) data regarding the relationship between ADHD and sleep disturbances. Another study comparing adults with ADHD to healthy control subjects demonstrated significantly increased nocturnal motor activity and more frequent arousals in the patients with ADHD but did not find differences in other polysomnography parameters, despite these patients with ADHD reporting subjectively worse sleep quality than controls [26]. A recent study by Sobanski et al demonstrated reduced sleep efficiency, longer sleep onset latency, more frequent nocturnal awakenings, and altered sleep architecture in adults with ADHD compared with controls [27].

In children with ADHD, subjective parental and patient reports of sleep disturbances are common [28]. Recent reports have linked ADHD in children to RLS, periodic limb movement disorder (PLMD), and sleep-disordered breathing [29-32]. Notably, however, objective sleep data have not demonstrated uniform results regarding sleep continuity or sleep architecture abnormalities in children with ADHD [33]. Moreover, there have been relatively few studies examining the effects of ADHD medications on sleep parameters in children with ADHD, and most evaluate the effects of short-acting stimulants [33].

Stimulants are the mainstay of pharmacologic therapy for adults with ADHD [2,4,34]. Approximately 7 million Americans filled at least one prescription for an ADHD therapy in 2007 [35]. Their effectiveness in reducing the symptoms of ADHD in adults is well documented [36-38]. Despite the lack of objective documentation of sleep difficulties, clinical studies commonly report sleep difficulties, namely delay of sleep onset and insomnia [12,28,34,39]. As stimulants wear off, they are sometimes associated with a rebound effect and a return of ADHD symptoms. Depending on the timing of the last dose of medication and the duration of effect for the drug, this can coincide with bedtime and may result in a delay of sleep onset [10,34,40]. However, the effects of stimulants on sleep patterns in patients with ADHD have not been fully elucidated [41], and it is clear that stimulants can cause insomnia and result in sleep disturbances in some patients [12,28,34,39].

There is some evidence that stimulants may improve sleep in patients with ADHD. In one study, after 3 weeks of treatment with stimulants, adults with ADHD demonstrated reduced nocturnal motor activity and improved sleep quality [25]. In another study, treatment with methylphenidate (MPH) resulted in significant reduction in sleep onset latency and improved sleep efficiency, but did not alter other parameters as measured by polysomnography [27]. Studies of sleep actigraphy measuring gross body movements over extended periods of time in adults with ADHD demonstrated that although adults treated with MPH experienced prolonged sleep onset latency and decreased total sleep time, they also experienced a decrease in nocturnal awakenings leading to more consolidated sleep reported as improved quality [42]. A recent study by Faraone et al suggests that neither once-daily OROS MPH nor transdermal MPH reliably caused sleep problems (or worsened existing sleep problems) in children with ADHD [43]. In another pediatric study, MPH significantly prolonged sleep-onset latency while atomoxetine decreased it [44]. In the same study, however, treatment with atomoxetine was associated with significantly more sleep interruptions than MPH [44]. So while it may be apparent that stimulants can cause sleep disturbances in some patients, the evidence is inconsistent.

Lisdexamfetamine dimesylate (LDX) is approved for the treatment of ADHD in children aged 6 to 12 years and in adults, and is the first prodrug stimulant. LDX is a therapeutically inactive molecule. After oral ingestion, LDX is converted to l-lysine and active d-amphetamine, which is responsible for the therapeutic effect. While a small amount of LDX is hydrolyzed to d-amphetamine in the gastrointestinal (GI) tract, the conversion of LDX into active d-amphetamine occurs primarily in the blood. The combination of l-lysine and d-amphetamine created a new chemical entity with a prodrug technology of delivery of d-amphetamine [45]. The absorption of LDX and its conversion to d-amphetamine are not affected by the pH of the GI system and is unlikely to be influenced by normal variations in GI transit times [46].

LDX has demonstrated efficacy in treating children and adults with ADHD in multiple randomized, double-blind, clinical trials. LDX has demonstrated significant efficacy over placebo in improving ADHD rating scale Version IV (ADHD-RS-IV-IV) scores, Swanson, Kotkin, Agler, M-Flynn, and Pelham (SKAMP) rating scale scores, and Permanent Product Measure of Performance (PERMP) scores compared with placebo in children with ADHD [47,48]. LDX was effective for up to 13 hours in a phase 3, randomized, controlled trial in children in a laboratory classroom study [49]. Adverse events (AEs) were consistent with other pediatric studies of LDX.

In a large, randomized, controlled trial in adults with ADHD, treatment with LDX resulted in significant improvements in ADHD-RS-IV and Clinical Global Impression (CGI) scores compared with placebo [50]. LDX was generally well tolerated in this study, and subjects reported common nonsleep-related adverse events including dry mouth and decreased appetite as well as insomnia. The results of this study were previously published. The present analysis, using the data-set from this previously published study [50], evaluated the effects of LDX on sleep parameters in adults with ADHD utilizing the Pittsburgh Sleep Quality Index (PSQI), a validated scale designed to measure overall sleep quality and related aspects of sleep [51]. This analysis was undertaken to describe the effects of LDX on sleep using the PSQI. Previously, the PSQI scale has not been extensively studied in adult patients with ADHD.

## Methods

This 4-week, double-blind, placebo-controlled, randomized, parallel-group, forced-dose, escalation study of LDX (Vyvanse^®^, Shire US Inc.) was conducted between May and November 2006. Institutional Review Board approval was obtained for each of the 48 study sites, and all participants gave written informed consent after receiving a full explanation of the study.

### Inclusion/Exclusion Criteria

Adults aged 18 to 55 years with a primary diagnosis of ADHD by *Diagnostic and Statistical Manual of Mental Disorders, Fourth Edition, Text Revision *(DSM-IV-TR) criteria were eligible for study enrollment. Investigators established a diagnosis of ADHD based on a psychiatric evaluation utilizing the Adult ADHD Clinical Diagnostic Scale. Participants were required to have clinician-rated ADHD-RS-IV scores of at least 28 at baseline using adult prompts developed by New York University and Massachusetts General Hospital [52,53]. This score indicates moderate to severe ADHD symptoms. Patients were excluded from the study if they had any comorbid psychiatric diagnosis with significant symptoms, hypertension, significant abnormality on electrocardiogram (ECG), history of seizures (other than infantile febrile seizures), or were significantly underweight or morbidly obese. Insomnia or sleep-related conditions were not listed as exclusion criteria. Additionally, subjects taking any medications that affect the central nervous system or blood pressure (excluding ADHD medications that were discontinued during a washout period) or those with a current or recent history of drug dependence or substance abuse (excluding nicotine) were also excluded from the study. Specifically, prohibited therapies relevant to this sleep analysis included sedatives, sedative-hypnotics such as zopiclone, sedating antihistamines (as a single preparation or in combination), anxiolytics, benzodiazepines, and benzodiazepine derivatives.

### Study Design and Measures

The present study had 3 phases: 1) screening and washout; 2) baseline; and 3) double-blind treatment. After a screening period to determine eligibility, subjects were asked to discontinue all ADHD medications for at least 7 days (28 days if the patient was taking atomoxetine). At the baseline visit, enrolled participants completed baseline assessments and were randomized to receive placebo or once-daily oral doses of LDX of 30 mg, 50 mg, or 70 mg during the 4-week, double-blind period. All subjects randomized to receive LDX began treatment with 30 mg doses. Subjects randomized to receive 50 mg/d LDX underwent a 1-week, forced-dose escalation schedule, while those randomized to the 70 mg/d LDX group underwent a 2-week escalation. During the 4-week, double-blind period, subjects attended weekly clinic visits for evaluation and drug distribution and were contacted within 30 days following their last dose of study medication to collect information about serious AEs. ADHD-RS-IV total score at endpoint was the primary efficacy measure in the present study.

Safety assessments included the recording of AEs, vital signs, ECGs, clinical laboratory tests, and physical examinations. AEs were collected at every study visit after screening and were coded using the *Medical Dictionary for Regulatory Activities *(MedDRA version 9.1. Reston, VA).

Sleep quality was assessed using the PSQI administered at baseline and at the endpoint (ie, week 4 or early termination). The PSQI consists of 19 self-rated questions and 5 questions intended to be completed by the subject's roommate or bed partner (if applicable) [51]. The 19 self-rated questions are grouped into 7 component scores: subjective sleep quality, sleep latency, sleep duration, habitual sleep efficiency, sleep disturbances, use of sleeping medications, and daytime dysfunction. Each component score ranges from 0 (no difficulty) to 3 (severe difficulty) and contributes equally to a PSQI global score that ranges from 0 to 21. Negative change scores indicate improvement on the PSQI. This validated instrument has been shown to be a sensitive and specific measure of sleep quality over a 1-month interval [51]. The PSQI underwent field testing, which demonstrated that the questionnaire is considered easy to use by subjects and patients. In addition, field testing showed that the 7 major components, as well as the 19 individual questions, are internally consistent. Responses for global scores, component scores, and individual question responses are stable across time, and its validity is supported by its ability to discriminate patients from controls [51]. Higher PSQI scores represent poorer sleep quality with global scores >5 generally considered to indicate poor sleep quality (diagnostic sensitivity of 89.6% and specificity of 86.5%) [51]. Furthermore, global scores >5 indicate that the subject is having severe difficulties in at least 2 areas, or moderate difficulties in more than 3 areas. The global score, thus, conveys information about the severity of the subject's sleep problems and the number of potential problems present [51].

### Data Analysis

The intent-to-treat (ITT) population, defined as all subjects randomized to treatment and who had both a baseline and at least 1 post-randomization ADHD-RS-IV total score available, was the primary population for efficacy analyses while safety analyses were performed on the safety population, defined as all subjects who received the study drug.

The primary efficacy analysis was performed on the change from baseline in ADHD-RS-IV total score at treatment endpoint, using a two-way analysis of covariance (ANCOVA). This method yielded results numerically identical to the last observation carried forward (LOCF) approach. To compare the ADHD-RS-IV change from baseline of the 3 LDX treatment groups with those patients receiving placebo, Dunnett's test for multiple mean comparisons with least-squares (LS) adjustment was used.

For safety analyses, the length of exposure to the study drug was categorized by week (ie, 1 week = 1 to 7 days, 2 weeks = 8 to 14 days, etc) and was determined by the dates of first dispensing and last dose of the study drug. AEs were reported as either prerandomization AEs or treatment-emergent AEs (TEAEs) based on the starting date of the AE in relation to the date randomized treatment was initiated. Events with missing start dates were considered TEAEs that began on the first date of treatment.

PSQI global and component scores were summarized by treatment for baseline and at endpoint (the final study visit) using the number of observations, mean, and standard deviation (SD). Changes in PSQI global and component scores from baseline were analyzed for differences at endpoint among treatment groups using the ANCOVA model. Dunnett's test for multiple mean comparisons with LS adjustment was utilized to compare the change from baseline of the 3 active treatment groups with the placebo group. Post hoc analyses included analyses of 2 questions in the PSQI requiring subjects to report time measures (sleep onset latency and sleep duration).

## Results

### Subject Demographics and Disposition

**Figure 1:**
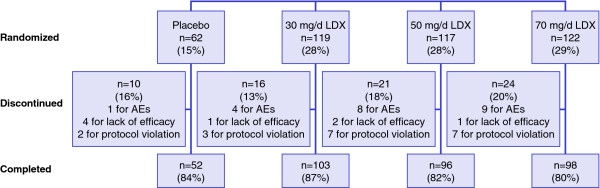
**Subject disposition**. (LDX = lisdexamfetamine dimesylate; AEs = adverse events).

The disposition of the 420 enrolled subjects is shown in Figure 1. The safety population was composed of the 420 participants and the ITT population consisted of 414 subjects (n = 115, 117, 120, and 62 for the 30 mg/d, 50 mg/d, 70 mg/d, and placebo groups, respectively). Of the enrolled subjects, 71 (17%) discontinued prior to study completion while 349 (83%) completed the study (Figure 1). For purposes of the PSQI, the safety population included only those patients at endpoint with a valid endpoint PSQI measurement (n = 402). Most subjects reporting sleep-related AEs while receiving LDX (103 of 109 subjects) or placebo (8 of 9 subjects) and all subjects (n = 9) discontinuing the study due to sleep-related AEs had both baseline and endpoint PSQI scores and were included in the analysis of sleep quality.

The treatment groups were well matched at baseline. Slightly more men than women were included in the safety population (between 51.6% and 56.4% were men across the groups). The mean age (SD) of the placebo group was 35.2 (10.9) years while the mean age in the LDX groups ranged from 34.2 (10.0) years to 35.8 (10.5) years. The mean (SD) weight of the placebo group was 181.3 (39.1) lb and the LDX groups ranged from 173.1 (37.8) lb to 178.1 (38.9) lb. The majority of subjects were Caucasian, making up 77.4% of the placebo group and 79.0% to 88.5% of the LDX groups. Disease severity, as assessed by ADHD-RS-IV total score and CGI-Severity, was similar among groups at baseline. Global PSQI scores at baseline were similar for all groups and indicated overall poor sleep quality with mean (SD) scores of 6.3 (3.20), 5.4 (2.53), 5.9 (3.01), and 6.0 (2.76) for the placebo, 30 mg/d LDX, 50 mg/d LDX, and 70 mg/d LDX groups, respectively. At baseline, 46.7%, 44.4%, 45.5%, and 57.3% of subjects in the placebo, 30 mg/d LDX, 50 mg/d LDX, and 70 mg/d LDX groups, respectively, rated their sleep as overall poor quality by global PSQI scores.

### Efficacy and Tolerability Findings from the Parent Study

Subjects receiving LDX showed significant improvement vs placebo in ADHD symptoms, measured by ADHD-RS-IV scores, and significant improvement in symptom severity vs placebo, measured by the Clinical Global Impressions-Improvement scale. AEs included dry mouth, decreased appetite, and insomnia; and were generally mild [50].

### Changes in PSQI Scores

**Figure 2:**
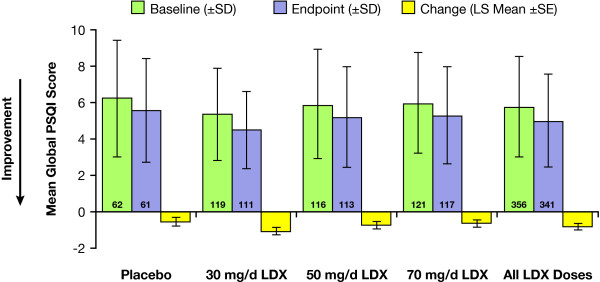
**Effect of LDX on global PSQI scores**. Mean global PSQI scores of all treatment groups decreased from baseline indicating an improvement in sleep quality. Numbers within bars indicate numbers of subjects. (SD = standard deviation; SE = standard error; LS = least squares; PSQI = Pittsburgh Sleep Quality Index; LDX = lisdexamfetamine dimesylate).

At endpoint, the mean global PSQI score, a composite of all component scores, for all treatment groups decreased from baseline (Figure 2). The global PSQI score LS mean (SE) change from baseline of the placebo group was -0.5 (0.26) and the combined LDX groups demonstrated LS mean (SE) changes of -0.8 (0.11) (*P *= .33). LDX treatment was not associated with a significant change from baseline compared with placebo indicating that there was no worsening of or impact on sleep quality with LDX. Further analyses employing Dunnett's test for multiple mean comparisons demonstrated that there were no significant differences in mean change from baseline at endpoint for any LDX dose group compared with placebo.

The mean changes from baseline at endpoint for the components of the PSQI, subjective sleep quality, sleep latency, sleep duration, habitual sleep efficiency, sleep disturbances, and use of sleep medication, were not significantly different between patients treated with any LDX dose and those receiving placebo.

The PSQI requires subjects to report average sleep onset (in minutes) and sleep duration (in hours) over the course of the month preceding the assessment. At baseline, the mean (SD) sleep onset latency for the placebo group was 30.2 (28.34) minutes compared with 24.8 (24.03) minutes for the combined LDX treatment group. Compared with placebo, LDX was not associated with significant prolongation of sleep onset (Table 1). In addition, LDX was not associated with significant changes in sleep duration from baseline at endpoint compared with placebo. LDX treatment was associated with change from baseline in sleep duration with LS mean (SE) of -0.2 (0.07) hour compared with -0.1 (0.15) for placebo (Table 2).

**Table 1:** Effect of LDX on Sleep Onset (in minutes) as Assessed by the PSQI

	**Placebo** **(n = 62)**	**30 mg/d LDX** **(n = 119)**	**50 mg/d LDX** **(n = 116)**	**70 mg/d LDX** **(n = 121)**	**All LDX Doses** **(n = 356)**
Baseline [Mean (SD)]	30.2 (28.34)	22.6 (20.84)	26.6 (26.44)	25.1 (24.56)	24.8 (24.03)

Endpoint [Mean (SD)]	28.1 (32.64)	20.1 (18.23)	27.9 (26.73)	29.3 (31.19)	25.9 (26.27)

Change [LS Mean (SE)]	-1.2 (2.78)	-3.5 (2.09)	0.4 (2.06)	4.0 (2.04)	0.4 (1.23)

Sleep onset reported in minutes.

SD = standard deviation; SE = standard error; LS = least squares; PSQI = Pittsburgh Sleep Quality Index; LDX = lisdexamfetamine dimesylate.

**Table 2:** Effect of LDX on Sleep Duration (in Hours) as assessed by the PSQI

	**Placebo** **(n = 62)**	**30 mg/d LDX** **(n = 119)**	**50 mg/d LDX** **(n = 116)**	**70 mg/d LDX** **(n = 121)**	**All LDX Doses** **(n = 356)**
Baseline [Mean (SD)]	6.9 (1.34)	7.4 (1.69)	7.4 (1.51)	7.2 (1.42)	7.3 (1.54)

Endpoint [Mean (SD)]	7.0 (1.33)	7.2 (1.21)	7.0 (1.44)	7.0 (1.61)	7.1 (1.43)

Change [LS Mean (SE)]	-0.1 (0.15)	-0.1 (0.11)	-0.3 (0.11)	-0.2 (0.11)	-0.2 (0.07)

SD = standard deviation; SE = standard error; LS = least squares; PSQI = Pittsburgh Sleep Quality Index; LDX = lisdexamfetamine dimesylate.

**Figure 3:**
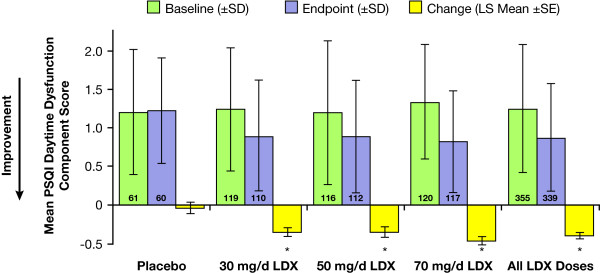
**Effect of LDX on daytime dysfunction (component 7) PSQI scores**. Mean PSQI component 7 score (daytime dysfunction component) decreased significantly after treatment with LDX. Numbers within bars indicate numbers of subjects. (SD = standard deviation; SE = standard error; LS = least squares; PSQI = Pittsburgh Sleep Quality Index; LDX = lisdexamfetamine dimesylate). *P*-values for "all LDX doses" calculated by analysis of covariance with baseline score as covariate. *P*-values for individual LDX dose groups calculated by Dunnett's test for multiple mean comparisons with least-squares (LS) adjustment. * *P *< .01 vs placebo.

Analysis of change from baseline in PSQI component scores demonstrated significant differences between treatment with LDX and placebo on the daytime dysfunction (trouble staying awake or lack of enthusiasm) component (ie, component 7) as shown in Figure 3. At endpoint, the LS mean (SE) changes from baseline of the PSQI daytime dysfunction score was 0.0 (0.08) for the placebo group and -0.4 (0.04) for the LDX all dose group (*P *= .0001). Significant improvements from baseline were evident for all individual LDX dose groups compared with placebo (*P *≤ .01 for each). The placebo-adjusted LS mean changes from baseline for the 30 mg/d, 50 mg/d, and 70 mg/d LDX groups were -0.3, -0.3, and -0.4, respectively.

### Concomitant Medications and Sleep-related Treatment-emergent Adverse Events

As previously mentioned, the use of sleep-inducing medications was prohibited in the protocol of this study. However, over the course of the trial, the use of sleep medications appeared evenly distributed across the dosage groups. Diphenhydramine and diphenhydramine hydrochloride were used by 10 subjects (placebo: 2; 30 mg/d LDX: 6; 50 mg/d LDX: 1; 70 mg/d LDX: 1). Some instances of diphenhydramine use were related to treatment of allergic reactions/poison ivy or to respiratory symptoms and not for treatment of sleep disorders. Zolpidem tartrate and melatonin were each used by 1 subject during the study (30 mg/d LDX and placebo group, respectively).

The incidences of all sleep-related TEAEs are listed in Table 3. The TEAEs with an incidence of at least 2% in any treatment group were initial insomnia (3.2%, 3.4%, 6.0%, and 5.7% for placebo, 30 mg/d, 50 mg/d, and 70 mg/d, respectively), insomnia (4.8%, 19.3%, 17.1%, and 21.3%, respectively), middle insomnia (0%, 4.2%, 1.7%, and 4.9%, respectively), somnolence (3.2%, 0.8%, 0%, and 0%, respectively), fatigue (4.8%, 7.6%, 4.3%, and 2.5%, respectively), and sleep disorder (3.2%, 0%, 1.7%, and 0%, respectively). Insomnia was reported as a TEAE by 14% (n = 50) of patients taking LDX during week 1, 2.6% (n = 9) in week 2, 3.1% (n = 10) in week 3, and 2.3% (n = 7) in week 4 and beyond. For patients receiving placebo, insomnia was reported by 3.2% (n = 2) during week 1, by 1.7% (n = 1) during week 2, and was not reported in later weeks. Sleep-related TEAEs in patients receiving LDX were generally mild or moderate in severity (Table 4). The only sleep-related TEAEs to be recorded as severe were insomnia in 2.2% of subjects receiving LDX (n = 8), fatigue in 0.6% of subjects in the LDX groups (n = 2) and nightmares in 0.3% of subjects in the LDX groups (n = 1). Treatment with LDX was not associated with any deaths or serious AEs in this study.

**Table 3:** Sleep-Related TEAEs

**n (%)**	**Placebo** **(n = 62)**	**30 mg/d LDX** **(n = 119)**	**50 mg/d LDX** **(n = 117)**	**70 mg/d LDX** **(n = 122)**	**Active Doses** **(n = 358)**
Initial insomnia	2 (3.2)	4 (3.4)	7 (6.0)	7 (5.7)	18 (5.0)

Insomnia	3 (4.8)	23 (19.3)	20 (17.1)	26 (21.3)	69 (19.3)

Middle insomnia	0 (0)	5 (4.2)	2 (1.7)	6 (4.9)	13 (3.6)

Somnolence	2 (3.2)	1 (0.8)	0 (0)	0 (0)	1 (0.3)

Sleep disorder	2 (3.2)	0 (0)	2 (1.7)	0 (0)	2 (0.6)

Abnormal dreams	0 (0)	0 (0)	0 (0)	1 (0.8)	1 (0.3)

Early morning awakening	0 (0)	0 (0)	0 (0)	1 (0.8)	1 (0.3)

Nightmare	0 (0)	0 (0)	0 (0)	2 (1.6)	2 (0.6)

Poor quality sleep	0 (0)	1 (0.8)	0 (0)	0 (0)	1 (0.3)

Hypersomnia	0 (0)	1 (0.8)	0 (0)	0 (0)	1 (0.3)

Fatigue	3 (4.8)	9 (7.6)	5 (4.3)	3 (2.5)	17 (4.7)

TEAEs = treatment-emergent adverse events; LDX= lisdexamfetamine dimesylate.

**Table 4:** Sleep-Related TEAEs on LDX by Severity

**Active doses (n = 358) n (%)**	**Mild**	**Moderate**	**Severe**
Initial insomnia	11 (3.1)	7 (2.0)	0 (0)

Insomnia	37 (10.3)	28 (7.8)	8 (2.2)

Middle insomnia	7 (2.0)	7 (2.0)	0 (0)

Somnolence	1 (0.3)	0 (0)	0 (0)

Sleep disorder	1 (0.3)	1 (0.3)	0 (0)

Abnormal dreams	1 (0.3)	0 (0)	0 (0)

Early morning awakening	1 (0.3)	0 (0)	0 (0)

Nightmare	1 (0.3)	0 (0)	1 (0.3)

Poor quality sleep	0 (0)	1 (0.3)	0 (0)

Hypersomnia	0 (0)	1 (0.3)	0 (0)

Fatigue	9 (2.5)	6 (1.7)	2 (0.6)

**Figure 4:**
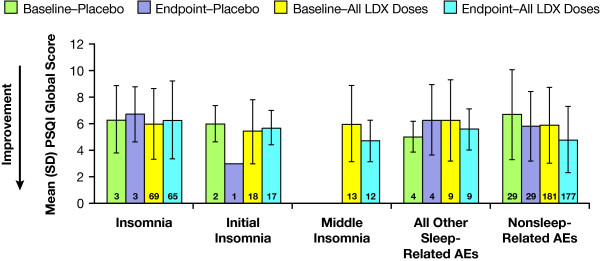
**PSQI global scores for subjects reporting sleep-related TEAEs**. PSQI scores are not worse at 4 weeks for subjects who reported sleep-related TEAEs. Numbers within bars indicate numbers of subjects. (TEAEs = treatment-emergent adverse events; PSQI = Pittsburgh Sleep Quality Index; LDX = lisdexamfetamine dimesylate; SD = standard deviation).

To investigate whether a relationship exists between the reported incidence of sleep-related TEAEs and subjective reports of sleep quality, we examined changes in PSQI scores of subjects reporting various sleep-related TEAEs. For subjects in all LDX treatment groups combined (n = 109) and in the placebo group (n = 9) who reported sleep-related TEAEs during the study, PSQI global scores from baseline to endpoint did not appear to appreciably worsen (Figure 4). For those subjects reporting TEAEs unrelated to sleep (n = 181 for all LDX treatment groups and n = 29 for placebo), there was a slight decline in PSQI scores from baseline to endpoint.

Nine subjects (3 in the 30 mg/d LDX group, 3 in the 50 mg/d LDX group, and 3 in the 70 mg/d LDX group) withdrew from the study due to sleep-related TEAEs. These 9 subjects reported a total of 10 sleep-related TEAEs (Table 5). Insomnia and other sleep-related TEAEs resulting in discontinuation were rated as mild to moderate for 7 of 10 TEAEs and as severe insomnia for the remaining 3 TEAEs. All but one case of insomnia were reported as resolved on follow-up. Discontinuations due to insomnia and sleep disorder did not demonstrate a discernible pattern with increasing LDX dose.

**Table 5:** Incidence of TEAEs Associated With Discontinuation

	**Placebo** **(n = 62)**	**30 mg/d LDX** **(n = 119)**	**50 mg/d LDX** **(n = 117)**	**70 mg/d LDX** **(n = 122)**	**Active Doses** **(n = 358)**
Discontinuation, n (%)					

Insomnia	0 (0)	2 (1.7)	3 (2.6)	3 (2.5)	8 (2.2)

Sleep disorder	0 (0)	0 (0)	1 (0.9)	0 (0)	1 (0.3)

Hypersomnia	0 (0)	1 (0.8)	0 (0)	0 (0)	1 (0.3)

## Discussion

The present analyses compared the effects of LDX and placebo on sleep characteristics in adults with ADHD. The data presented in this manuscript were collected as part of a phase 3 trial in which LDX demonstrated significant efficacy at reducing the symptoms of ADHD in adults compared with placebo. To assess sleep parameters, subjects were asked to complete the PSQI, a validated measure of sleep quality, before treatment and again at the end of a 4-week, double-blind treatment period or early termination, during which they were treated with placebo or any of 3 LDX dosages. Additional sleep characteristics were assessed by the analysis of sleep-related TEAEs.

Mean baseline global PSQI scores for all treatment groups were greater than 5, confirming the results of previous studies demonstrating that adults with ADHD report poor sleep quality [8]. LDX was not associated with an overall worsening of self-reported sleep quality. There was also no difference or worsening between the LDX and placebo groups with reference to the PSQI components of sleep latency, sleep duration, sleep disturbances, sleep medications used, and habitual sleep efficiency. Furthermore, LDX was not associated with significant alterations in sleep duration or sleep onset latency compared with placebo. PSQI global scores decreased in all groups in the direction of improvement, and these differences were not statistically significant for LDX or placebo groups.

Analysis of individual PSQI components showed that LDX significantly improved daytime functioning compared with placebo. The PSQI assesses daytime functioning by asking subjects to report 1) how often they have trouble staying awake during activity and 2) how much of a problem they have keeping up enough enthusiasm to get things done [51]. While the PSQI daytime functioning component scores were statistically improved vs placebo and the overall group change from baseline for the LDX group on this component was ~30% (baseline mean of 1.25, endpoint mean of 0.87), the clinical significance of this finding was difficult to assess since global PSQI scores and other component scores were not significantly improved. The PSQI component that assesses subject's self-rating of sleep quality showed that, regardless of treatment group, subjects rated their sleep quality at endpoint as "fairly good" (mean scores of 1.03 and 0.97 for placebo and combined all-active LDX treatment groups, respectively).

Previous research has demonstrated a significant correlation between the severity of ADHD symptoms and parameters of sleep quality. Schredl et al, using 2 sleep questionnaires (Schlaffragebogon B [SF-B] and Landexker Inventar zur Erfassung con Schlafstörungen [LISST]), demonstrated that ADHD symptoms were inversely and closely related to the feeling of being refreshed in the morning, and directly related to problems with sleep/wake pattern, sleep quality, sleep latency, and tiredness during the day [24]. The positive effect of LDX on a subjective measure of daytime function and other data suggestive of potentially broader positive effects on subjective sleep measures in the present study are intriguing. In line with this, other preliminary reports have described positive effects of stimulants on sleep quality in adults with ADHD. In an open-label study of 8 adults with ADHD, Kooij et al demonstrated that stimulants improved self-reported sleep quality [25]. Recently, in a small, open-label study, Sobanski et al found that stimulant therapy was associated with significant improvements in the restorative value of sleep as assessed by Schlaffragebogon A (SF-A) [27]. Taken together, these findings indicate that more studies that rigorously assess sleep quality with and without treatment in adults with ADHD are needed.

Insomnia, middle insomnia, and initial insomnia were reported as AEs more commonly in patients receiving LDX than those patients receiving placebo. This reported incidence of insomnia markedly decreased after week 1, which cannot be accounted for by study discontinuations. Furthermore, most sleep-related TEAEs were mild or moderate in severity and rarely resulted in subjects being discontinued from the trial. Interestingly, despite reports of sleep-related TEAEs, global PSQI scores did not appear to worsen in those subjects at the end of treatment. This raises the possibility that frequency counts of AEs may not be a strong predictor of qualitative, subject-reported clinical consequences in this setting. The overall incidence of sleep-related TEAEs in the present study compares favorably with data for other medications used to treat ADHD in adults. In 1 study, insomnia was reported by 37% of adult patients receiving mixed amphetamine salts during a 7-week trial and studies of atomoxetine, a nonstimulant, reported insomnia as a TEAE in 20.8% of adult subjects during 2 10-week trials [37,54].

Interestingly, despite insomnia being reported as a frequent TEAE in patients receiving LDX, PSQI results indicated that LDX was not associated with a worsening in sleep duration, sleep onset latency, or sleep quality compared with patients taking placebo. This suggests that although insomnia was reported as an AE in this trial, subjects did not feel it interfered with their overall sleep quality. A notion that is also supported by the finding that for those subjects who reported insomnia as an AE, there was no worsening of global PSQI scores (see Figure 4). On the other hand, that the subjects reporting TEAEs unrelated to sleep showed a slight improvement in PSQI scores, raises the possibility that in the absence of TEAEs that affect sleep, PSQI-measured sleep quality may improve with treatment. Since sleep-related TEAEs did occur in some subjects while overall sleep quality by group did not worsen, the possibility also exists that improvement in the daytime functioning component of the global PSQI rating may have masked deterioration in other components of sleep quality for some individuals. When considering the significance of such correlations, however, it is important to keep in mind that AEs were collected as spontaneous reports weekly and measured discrete, new events with an onset in the preceding week, while the PSQI is designed to assess sleep quality during the month preceding assessment [51].

This study has several limitations. Adults with comorbid psychiatric disease were excluded from the current study, potentially limiting generalization of these results to the broader adult population with ADHD. Additionally, subjects with severe pre-existing insomnia may not have chosen to participate in this study due to the potential for stimulants to affect sleep. This study was designed as a placebo-controlled, forced dose-titration trial, and, therefore, no active comparator was employed and subjects may not have been receiving a dose of LDX that provided optimal balance between efficacy and tolerability. The current study assessed sleep parameters using the PSQI, a self-report measure that is potentially affected by subject-specific inaccuracies in reporting sleep characteristics. Further research with sleep parameters as a primary objective and using optimized dose design and polysomnography or actigraphy, which would allow for more objective sleep measurements, would be useful in further characterizing the potential for detrimental effects of long-acting stimulants on sleep. Despite these limitations, this study provides further evidence that LDX demonstrated a safety profile similar to other stimulant medications for the treatment of adults with ADHD with no overall worsening of sleep quality.

## Conclusion

In this population of adults with ADHD, sleep quality at baseline, as assessed by the PSQI, was generally poor. In those subjects who reported sleep-related TEAEs, PSQI scores were no different from placebo. These findings highlight the importance of relying on data beyond the frequency count of insomnia and other sleep-related AEs in determining the potential clinical relevance of adverse effects of stimulants on sleep parameters. LDX was found to significantly improve self-reported daytime functioning and, despite reports of insomnia as a TEAE, treatment with LDX was not associated with an overall worsening of self-reported sleep quality, sleep onset latency, sleep duration, or sleep disturbances. Sleep-related TEAEs associated with LDX treatment were generally mild or moderate in severity, led to few discontinuations, and decreased incidence after the first week of treatment. These results suggest that the global sleep quality of subjects on LDX and placebo is no different and that daytime functioning in adults with ADHD on LDX significantly improves. To improve compliance, treatment of adults with ADHD in clinical practice may be enhanced by closer monitoring of patients for sleep-related AEs. Perhaps including an assessment tool such as the PSQI, which complements spontaneously reported sleep-related AEs in clinical practice, may also contribute to improved outcomes and compliance.

## Declaration of competing interest

Dr Adler has received royalty payments as an inventor from New York University. He receives/d grant research support from Abbott Laboratories, Bristol-Myers Squibb, Cortex Pharmaceuticals, Merck & Co., Inc., Novartis Pharmaceuticals Corporation, Pfizer Inc, Shire, Eli Lilly and Co., Ortho-McNeil/Janssen/Johnson&Johnson, New River Pharmaceuticals, Cephalon Inc, and National Institute of Drug Abuse. He is/has been a speaker for Eli Lilly and Co. and Shire. He is/has been on the advisory board and consulted for Abbott Laboratories, Cortex Pharmaceuticals, Novartis Pharmaceuticals Corporation, Pfizer Inc, Shire, Eli Lilly and Co., Ortho-McNeil/Janssen/Johnson&Johnson, New River Pharmaceuticals, Cephalon Inc, Merck & Co., Inc., Organon, Sanofi-Aventis Pharmaceuticals, Psychogenics, and Mindsite.

Dr Goodman receives/d research support from Forest Labs, Shire Inc., McNeil, Cephalon, New River Pharmaceuticals, and Lilly and Company. He is/has received honoraria from Forest Labs, Lilly and Company, Shire Inc., McNeil, and Wyeth; is/has been a speaker for Forest Labs, Shire Inc., McNeil, and Wyeth; is/has been a consultant for Forest Labs, Lilly and Company, Shire Labs, McNeil, New River Pharmaceuticals, Thompson Reuters, and Clinical Global Advisors; receives royalties from MBL Communications: and is an equity shareholder in Wyeth.

Dr Weisler receives/d research support from the National Institute of Mental Health, Pfizer, Lilly, GlaxoSmithKline, Abbott, Merck, Organon, Bioavail, Shire, Sanofi-Synthelabo, AstraZeneca, Janssen, Wyeth Ayerst, Solvay, Novartis, Schwabe/Ingenix, Bristol-Myers Squibb, TAP Pharmaceutical, Synaptic Pharmaceutical Incorporated, Eisai, UCB Pharma, Inc., Cephalon, Lundbeck, Forest, Pharmacia, Neurochem, Cenerx, Eli Lilly, Ciba-Geigy, Vela, Parke Davis, Sandoz, Upjohn, MediciNova, SmithKline Beecham, Saegis, Corcept, New River Pharmaceuticals, McNeil, Burroughs Wellcome, CoMentis, Johnson & Johnson, Takeda, Sepracor, Repligen, and Dainnpon-Sumitomo. He is/has been a speaker for Wyeth Ayerst, Solvay, GlaxoSmithKline, Lilly, AstraZeneca, Cephalon, Validus, Bioavail, Abbott, Forest, Bristol-Myers Squibb, Shire, Pfizer, Eli Lilly, Organon, and Sanofi. He is/has been a consultant for ATSDR (Agency for Toxic Solvent Disease Registry), CDC (Centers for Disease Control), Bristol-Myers Squibb, Bioavail, Lilly, Wyeth Ayerst, Shire, Organon, Sanofi-Synthelabo, Forest, Solvay, Johnson & Johnson, Novartis, Ostuka America Pharma, Corcept, Abbott, Pfizer, GlaxoSmithKline, and Validus. He is/has been a financial stockholder of Merck, Pfizer, Bristol-Myers Squibb, Cortex, Abbott, and Johnson & Johnson.

Mr Hamdani is an employee of Shire Development Inc.

Dr Roth receives/d research grant support from Aventis, Cephalon, GlaxoSmithKline, Neurocrine, Pfizer, Sanofi, Schering Plough, Sepracor, Somaxon, Syrex, Takeda, TransOral, Wyeth, and Xenoport. He is/has been a speaker for Cephalon, Sanofi, and Takeda. He is/has been a consultant for Abbott, Accadia, Acoglix, Actelion, Alchemers, Alza, Ancil, Arena, AstraZeneca, Aventis, AVER, Bristol-Myers Squibb, BTG, Cephalon, Cypress, Dove, Elan, Eli Lilly, Evotec, Forest, GlaxoSmithKline, Hypnion, Impax, Intec, Intra-Cellular, Jazz, Johnson & Johnson, King, Ludbeck, McNeil, MediciNova, Merck, @Neurim, Neurocrine, Neurogen, Novartis, Orexo, Organon, Prestwick, Procter and Gamble, Pfizer, Purdue, Resteva, Roche, Sanofi, Schering Plough, Sepracor, Servier, Shire, Somaxon, Syrex, Takeda, TransOral, Vanda, Vivometrics, Wyeth, Yamanuchi, and Xenoport.

## Authors' contributions

LAA was the principal investigator on this study, made substantial contributions to the conception and design of the study, enrolled patients, participated in data acquisition, analysis, interpretation, and presentation. He was deeply involved in drafting the manuscript and revising the intellectual content. He has given final approval of this version. DG was an investigator on the parent study, enrolled patients, and participated in data acquisition, analysis, interpretation, and presentation. He was deeply involved in drafting the manuscript and revising the intellectual content. He has given final approval of this version. RW was an investigator on the parent study, enrolled patients, and participated in data acquisition, analysis, interpretation, and presentation. He was deeply involved in drafting the manuscript and revising the intellectual content. He has given final approval of this version. MH was the statistician for this study and made substantial contributions to the analysis and interpretation of the data. He was deeply involved in drafting the manuscript and revising the intellectual content. He has given final approval of this version. TR is a key expert in the area of sleep medicine and was deeply involved in the analysis and interpretation of the data and in the drafting and revising of the manuscript, making substantive contributions to all important intellectual content. He has given final approval of this version.
